# Reanalysis of Immunopeptidomics Datasets Provides Mechanistic Insight into TAPBPR-Mediated Peptide Editing on HLA-A, -B and -C Molecules

**DOI:** 10.12688/wellcomeopenres.20738.2

**Published:** 2025-11-25

**Authors:** Arwen F Altenburg, Jack L Morley, Sophia M Decatris, Jens Bauer, Juliane S Walz, Louise H Boyle

**Affiliations:** 1 Department of Pathology, University of Cambridge, Cambridge, England, CB2 1QP, UK; 2 Department of Peptide-based Immunotherapy, Institute of Immunology, University and University Hospital, Tübingen, Germany; 3 Cluster of Excellence iFIT (EXC2180) “Image-Guided and Functionally Instructed Tumor Therapies”, University of Tübingen, Tübingen, Germany; 4 Clinical Collaboration Unit Translational Immunology, German Cancer Consortium (DKTK), Department of Internal Medicine, University Hospital Tübingen, Tübingen, Germany

**Keywords:** TAPBPR, HLA-I, antigen presentation, peptide editing, immunopeptidomics, editing loop, molecular dynamic simulation

## Abstract

**Background:**

Major histocompatibility class I (MHC-I, human leukocyte antigen [HLA] class I in humans) molecules present small fragments of the proteome on the cell surface for immunosurveillance, which is pivotal to control infected and malignant cells. Immunogenic peptides are generated and selected in the MHC-I antigen processing and presentation pathway. In this pathway, two homologous molecules, tapasin and TAPBPR, optimise the MHC-I peptide repertoire that is ultimately presented at the plasma membrane. Peptide exchange on HLA class I by human TAPBPR involves the flexible loop region K22-D35, with the leucine at position 30 (L30) involved in mediating peptide dissociation. However, our understanding of the exact molecular mechanisms governing TAPBPR-mediated peptide exchange on HLA class I allotypes remains incomplete.

**Methods:**

Here, in-depth re-analyses of published immunopeptidomics datasets was used to further examine TAPBPR peptide editing activity and mechanism of action on HLA class I. The role of the TAPBPR editing loop in opening the HLA class I peptide binding groove was assessed using molecular dynamics simulations and a peptide exchange assay.

**Results:**

We show that TAPBPR shapes the peptide repertoire on HLA-A, -B and -C allotypes. The TAPBPR editing loop was not essential to allow HLA class I to adopt an open state but did allow for the HLA-A*68:02 peptide binding groove to stay open for a sustained period. L30 in the TAPBPR editing loop was typically sufficient to mediate peptide repertoire restriction on the three HLA class I allotypes expressed by HeLa cells. TAPBPR was also able to load peptides onto HLA class I in a loop-dependent manner.

**Conclusions:**

These results suggest that the TAPBPR editing loop is involved both in peptide filtering and loading.

## Plain Language Summary

Major histocompatibility complex (MHC) class I molecules play an essential role in alerting the immune system to infection and cellular changes. They do this by displaying small fragments of proteins (peptides) from pathogen-infected cells and tumours on the cell surface to immune cells. When activated, immune cells can then destroy the target cell. In 2015, we discovered that a novel accessory protein, called TAPBPR, assists in the selection of peptides displayed on MHC class I molecules for immune surveillance. A specific region in the TAPBPR protein – the editing loop – is known to be involved in removing peptides from MHC class I. However, our understanding of the process of peptide selection on MHC class I molecules remains incomplete. Here, we show that TAPBPR is not only involved in removing peptides from MHC class I molecules but also assists in peptide loading. Additionally, we demonstrate that the TAPBPR editing loop is involved in both removing and loading of peptides. Our results suggest that TAPBPR fine-tunes the peptide repertoire displayed on three different types of MHC class I molecules. Developing our understanding of the mechanisms of peptide selection on MHC class I molecules has important implications in disease and the development of new therapies.

## Introduction

Immunogenic peptide presentation on major histocompatibility class I (MHC-I) molecules (human leukocyte antigen [HLA] class I in humans) is essential for induction of cytotoxic CD8 ^+^ T cell responses against infected or malignant cells. Peptides are typically generated by proteasomal degradation and selected in the MHC-I antigen processing and presentation pathway. In this pathway, two homologous peptide editors, tapasin and TAPBPR, control and optimise the peptide repertoire that is ultimately presented at the cell surface. Tapasin functions as part of the multiprotein peptide loading complex (PLC) containing MHC-I, the peptide transporter associated with antigen processing (TAP), calreticulin, and ERp57 ^1, 2^. Interactions within the PLC stabilises MHC-I in a peptide-receptive state and allows tapasin to load peptide on MHC-I ^3– 6^. More recently the tapasin-related molecule TAPBPR was discovered, which functions outside the PLC ^7– 10^. TAPBPR can directly catalyse peptide exchange on MHC-I and can recruit UDP-glucose:glycoprotein glucosyltransferase 1 (UGT-1), which subsequently reglucosylates peptide-receptive MHC-I to recycle it back to the PLC for tapasin-mediated peptide acquisition ^11– 13^.

HLA class I molecules are highly polymorphic, and it has previously been shown that some allotypes are more dependent on tapasin than others to acquire peptide ^3, 14, 15^. Allotypes independent of tapasin generally present a broader repertoire ^16^. While it has yet to be determined whether HLA class I molecules display any TAPBPR-dependency, TAPBPR binds more strongly to HLA-A compared to HLA-B and -C molecules ^17^. Members of the HLA-A*02 and -A*24 superfamily are particularly susceptible to TAPBPR-mediated peptide editing in assays using recombinant TAPBPR ^17^. Interestingly, allotypes that were identified as strong TAPBPR binders were previously identified as tapasin-independent ^16, 17^, suggesting there may be an inverse correlation between tapasin and TAPBPR-dependence in the generation of optimally loaded peptide-HLA class I complexes ^18^.

Several structures of MHC-I in complex with peptide editors have provided insight into the mechanism of action (reviewed in 19, 20). TAPBPR has been suggested to widen and stabilise the peptide binding groove to facilitate peptide exchange ^21, 22^. Molecular dynamics (MD) simulations indicated that TAPBPR can remodel the B pocket of empty MHC-I, causing the peptide binding cleft to enter a more peptide-receptive conformation and facilitating the binding of peptide ^23^. A flexible loop region spanning residues K22-D35 has been identified in TAPBPR that resides at the interface of the MHC-I peptide binding groove ^22^. It was proposed that this editing loop acts as a *scoop loop* and occupies the MHC-I F pocket in the peptide binding groove, where the C-terminus of a bound peptide would reside ^22, 24^. The leucine at position 30 (L30) in the TAPBPR editing loop was shown critical for peptide dissociation, potentially through interaction with the F pocket ^25^. It was suggested that this *leucine lever* in the MHC-I peptide binding groove can only be outcompeted by a high affinity peptide, thereby filtering and optimising the peptide repertoire ^22, 24^. Alternatively, it has been proposed that the TAPBPR editing loop does not enter the MHC-I peptide binding groove but instead hovers above it to act as a *peptide trap* to promote peptide loading ^26^. This was supported by MD simulations showing that the peptide C-terminus may be drawn into the F pocket by the arginine at position 27 (R27) in the TAPBPR K22-D35 loop interacting with the carboxyl group of a peptide ^23^. These two seemingly contradicting hypotheses highlight that our understanding of the mechanisms of TAPBPR-mediated MHC-I peptide editing is incomplete.

Editing of the HLA class I peptide repertoire can experimentally be addressed using the mass spectrometry-based approach immunopeptidomics. This technique has been instrumental to enhance our understanding of TAPBPR as an HLA class I peptide editor, to explore the role of the TAPBPR-UGT-1 interaction and to establish the importance of the editing loop ^9, 12, 25^. For example, Hermann *et al.* used immunopeptidomics to show TAPBPR-mediated restriction of the peptide repertoire on HLA-A and -B molecules ^9^. Furthermore, Ilca *et al.* produced immunopeptidomics datasets to explore the contribution L30 in the editing loop to TAPBPR-mediated peptide dissociation ^25^. However, the datasets in these two publications also contained a wealth of additional information that remained unexplored. Here, we re-examine these published immunopeptidomics datasets to obtain a more comprehensive understanding of TAPBPR-mediated peptide selection on HLA class I. We show that TAPBPR shapes the peptide repertoire on HLA-A, -B and -C molecules, with the editing loop not only involved in filtering but also loading of peptides. While L30 was typically sufficient to mediate peptide filtering, we show additional or other residues in the TAPBPR editing loop likely contribute to peptide loading onto HLA class I.

## Methods

### HLA class I peptide analyses

**Figure 1.**
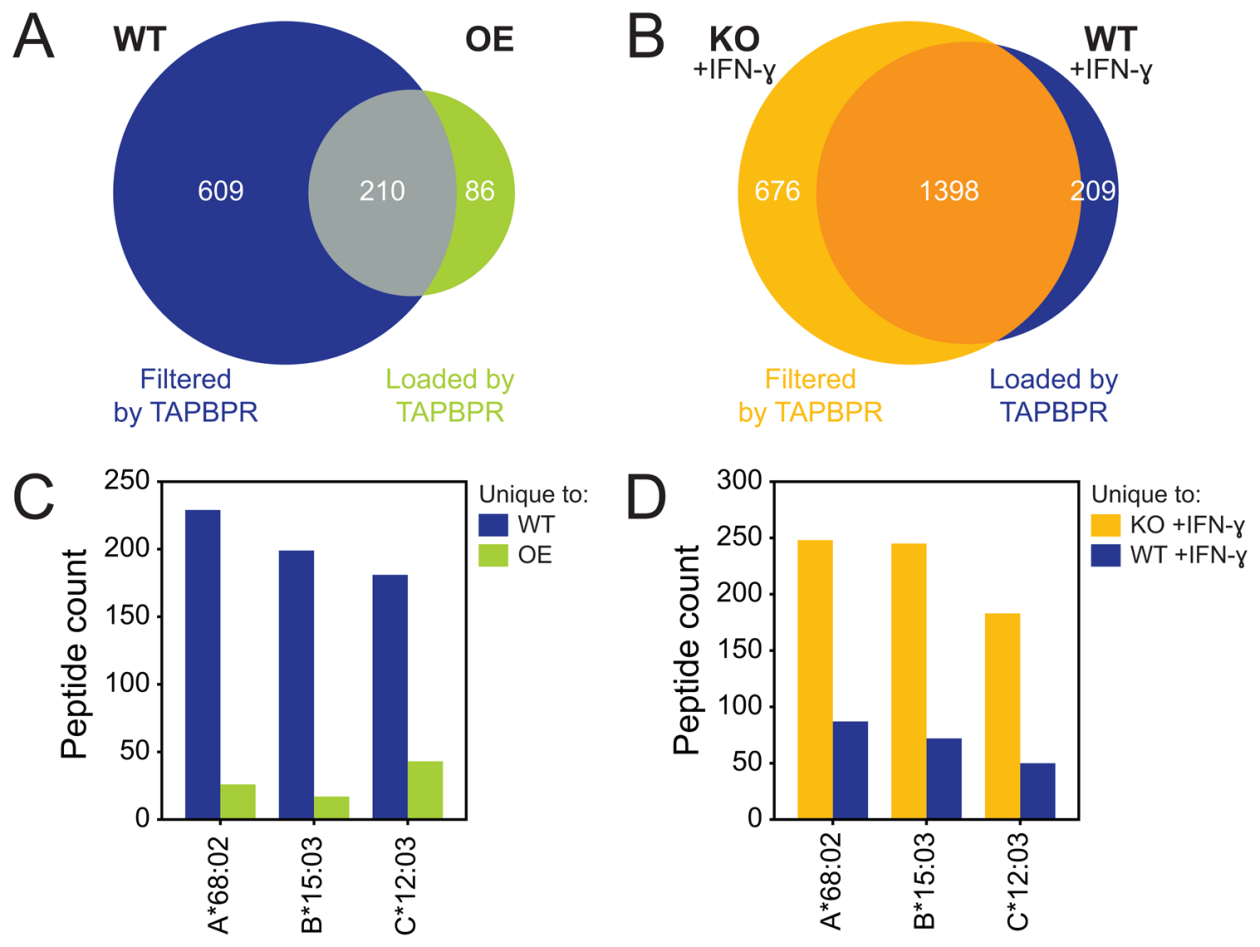
TAPBPR restricts the peptide repertoire diversity on HLA-A*68:02, B*15:03 and C*12:03. Peptides were eluted from W6/32-reactive HLA complexes isolated from ( **A**/ **C**) wild-type (WT) HeLaM and cells transduced to overexpress (OE) TAPBPR, or ( **B**/ **D**) HeLaM TAPBPR knock-out (KO) cells and WT cells treated with IFN-γ to induce endogenous TAPBPR expression ^9^. ( **A**– **B**) Size and overlap of the eluted peptide repertoire. ( **C**– **D**) NetMHC was used to assign the peptides to one of the three HLA class I allotypes expressed by HeLa cells ^9^. The absolute peptide count per HLA class I allotype was shown.

**Figure 2.**
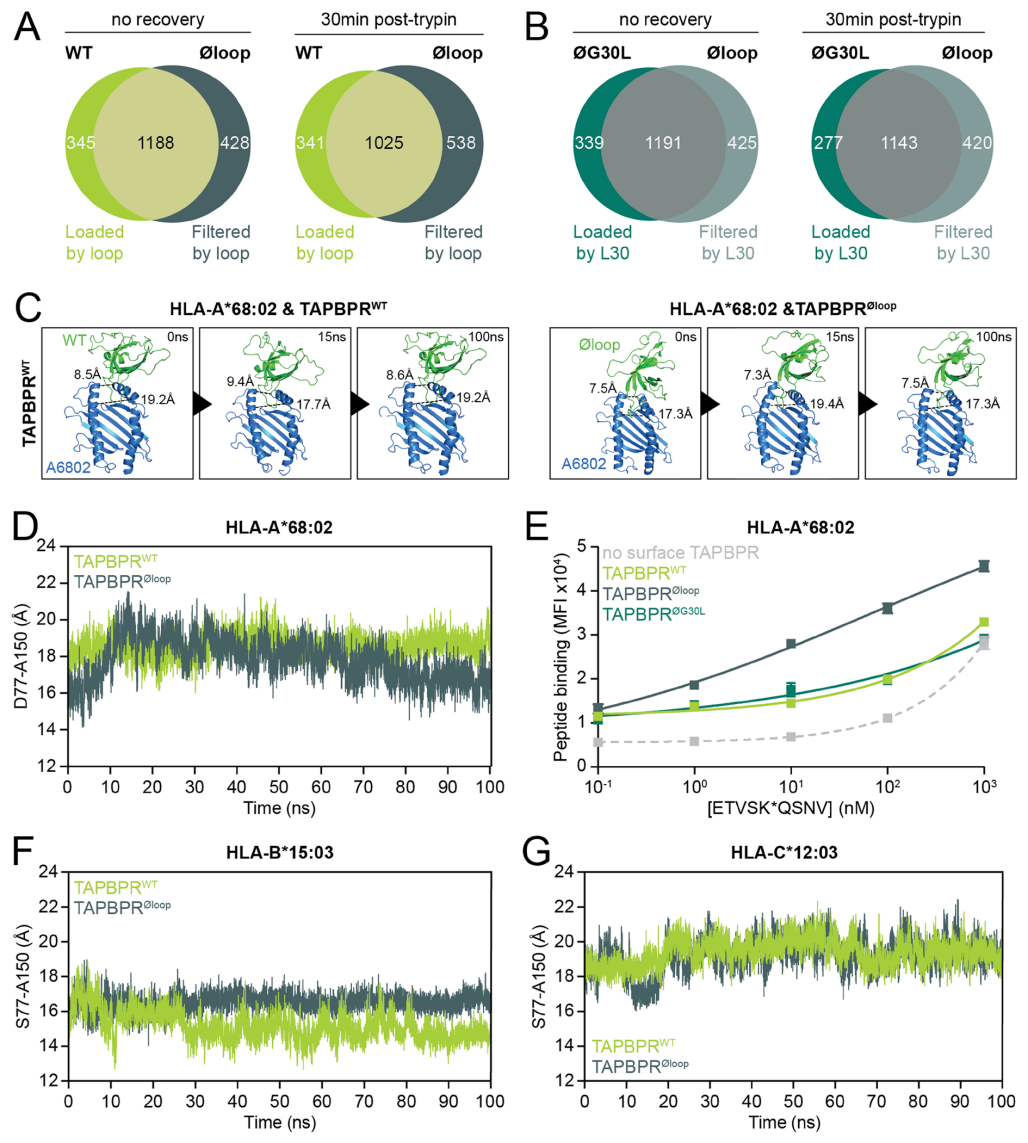
L30 in the TAPBPR editing loop restricts the HLA class I peptide repertoire diversity on HeLa cells. ( **A**– **B**) IFN-γ-treated HeLaM TAPBPR KO cells that were transduced to express TAPBPR ^WT^, TAPBPR ^øloop ^(mutated residues of the editing loop domain), or TAPBPR ^øG30L^ (TAPBPR ^øloop^ in which the leucine had been reconstituted at position 30) were harvested immediately post-trypsinisation (no recovery) or allowed to recover for 30min at 37°C in warm media (30min post-trypsin) ^25^. Subsequently, peptides were eluted from W6/32-reactive HLA complexes. Size and overlap of the eluted peptide repertoire are shown. ( **C**) Sort-time MD simulation of TAPBPR ^WT^ or TAPBPR ^øloop^ in complex with peptide-deficient HLA-A*68:02 at 0, 15 and 100ns. The distance between the HLA class I α1 and α2 helix was measured at Y84-A139 (top dotted line) and D77-A150 (dotted line in the middle of the groove) to show opening of the HLA class I peptide binding groove. ( **D**) The distance between the HLA-A*68:02 α1 and α2 helix measured from D77 to A150 during the 100ns MD simulation. ( **E**) Dose-response curves showing the level of exogenous ETVSK*QSNV (* indicates TAMRA label) peptide binding to HeLaM HLA-ABC ^KO^ cells reconstituted with HLA-A*68:02 and transduced to overexpress the indicated TAPBPR variants. Error bars represent SEM from 2 technical repeats. Data is representative of three independent repeats. ( **F**– **G**) The distance between the peptide-deficient HLA-B*15:03 (F) or -C*12:03 (G) α1 and α2 helix measured from S77-A150 during 100ns MD simulations.

**Figure 3.**
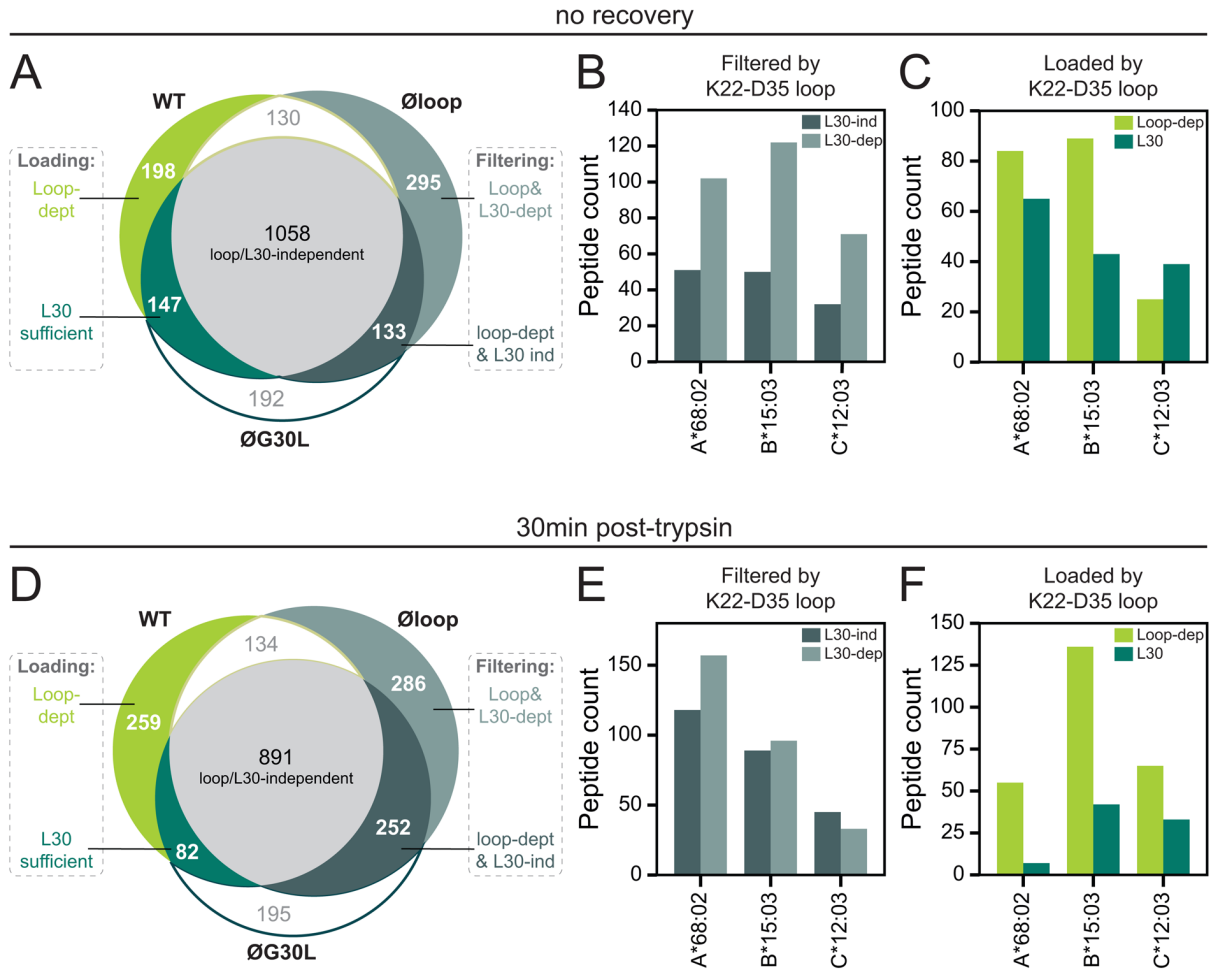
Different residues are involved in TAPBPR editing loop-mediated peptide filtering and loading. Cells were harvested immediately post-trypsinisation (no recovery, **A**– **C**) or allowed to recover for 30min at 37°C in warm media (30min post-trypsin, **D**– **F**). Peptides were eluted from W6/32-reactive HLA complexes isolated from IFN-γ-treated HeLaM TAPBPR KO cells that were transduced to express TAPBPR ^WT^, TAPBPR ^øloop^ (mutated residues of the editing loop domain), or TAPBPR ^øG30L ^(TAPBPR ^øloop^ in which the leucine had been reconstituted at position 30) ^25^. ( **A**/ **D**) Size and overlap of the peptide repertoire. ( **B**– **C**/ **E**– **F**) NetMHCpan-4.0 was used to assign peptides to one of the three HLA class I allotypes expressed by HeLa cells ^25^. The absolute peptide count per HLA class I allotype from each of the subgroups indicated in **A**/ **D** was shown.

HeLaM cells (a variant of the HeLa cell line that is more responsive to interferons ^27^), either wild-type, depleted of TAPBPR or transduced to overexpress (OE) TAPBPR, were described previously ^7, 9^. HeLaM cells depleted of TAPBPR and transduced to express TAPBPR K22-D35 loop mutants were previously published ^25^. Where indicated, cells were treated with 50 U/ml of IFN-γ (Peprotech, #300-02) at 37°C for 48–72h to induce endogenous TAPBPR expression. HLA class I expression levels were confirmed in the original publications ^25, 28^. Cells were harvested (without or with 30min post-trypsin recovery in media at 37°C), followed by lysis and HLA class I-peptide complex affinity purification, ligandome detection by mass spectrometry, and initial dataset processing as previously published ^9, 25^. LC-MS/MS measurements related to Figure 1 were conducted with three technical replicates applying a data-dependent acquisition method. Binder prediction was conducted with the online version of NetMHC-3.0 ^29^. Peptide elution experiment related to Figure 2 & Figure 3 were performed in five technical replicates for all cell lines, as described in the original publication ^25^. HLA annotation was performed using NetMHCpan-4.0 ^30^ with a percentile threshold of 2%. Peptide lists of predicted binders were further processed with original Python scripts using NumPy ^31^, pandas ^32^, matplotlib ^33^, and seaborn ^34^ packages.

### MD simulations

Structures of HLA-A*68:02, -B*15:03 and -C*12:03 were generated using their respective sequences from the IPD-IMGT/HLA database ^35^ and homology modelling using SWISS-MODEL ^36^. TAPBPR ^øloop^ was generated by the Rotamer tool of UCSF Chimera ^37^, with each most probable rotamer (as determined by the Dunbrack library ^38^) implemented into the loop. The mutated residue and those within 5Å underwent 100 steps of energy minimization using an AMBER ff14SB forcefield ^39^. The three-dimensional structure of the TAPBPR:MHC-I complex was obtained from PDB 5OPI ^22^. Structures were aligned to 5OPI to generate the TAPBPR:HLA class I complexes. All-atom MD simulations were carried out with GROMACS v2021.3 and v2021.5 ^40^. Simulations were carried out under the AMBER99SB-ILDN ^41^ protein forcefield and TIP3P ^42^ water model. SETTLE ^43^ and LINCS ^44^ constraint algorithms were applied to constrain water and protein molecules, respectively. Systems were solvated to an overall neutral charge containing Na ^+^ and Cl ^-^ ions to yield a physiological concentration of 0.15M. Short range interactions were treated with a Verlet cut-off scheme, while van der Walls forces and long-range electrostatics were handled with the Particle Mesh Ewald method ^45^ with grid spacing of 1.2Å and cubic interpolation. A velocity-rescaling thermostat ^46^ was used to maintain temperature constant at 300K with a coupling time constant of 0.1ps, using a thermodynamic ensemble of nPT. An isotropic Berendsen ^47^ or Parrinello-Rahman barostat was used to maintain a constant 1.0 bar pressure, with a coupling time constant of 0.5ps and 4.5×10 ^-5^ bar ^-1^ compressibility. Simulations were run for 100ns. For the simulations with HLA-B*15:03 or -C*12:03, the periodic boundary condition (PBC) jumps were removed, whole molecules were reconstructed, and the protein complex was centred. MD simulations were analysed using the PyMOL Molecular Graphics System (version 3.0, Schrödinger, LLC) and Visual Molecular Dynamics 1.9 ^48^.

### Cell culture

HeLaM and HEK293T cells (both gifts from Paul Lehner, University of Cambridge) were cultured in Dulbecco’s Modified Eagle’s medium (DMEM; Gibco, 41966052), supplemented with 10% foetal bovine serum (FBS; Gibco, 10500064), 100 U/ml penicillin and 100 mg/ml streptomycin (Gibco, 15140122). Cell lines were maintained at 37°C and 5% CO _2_.

### Lentiviral transduction

HEK293T cells were transfected with 0.8μg TAPBPR ^WT^, TAPBPR ^øloop^, or TAPBPR ^øG30L^ in pHRSIN-C56W-UbEM ^25, 49^, 0.5μg packaging vector pCMVΔR8.91 and 0.5μl envelope vector pMD.G. Supernatant containing lentivirus was harvested at 48 and 72 hours following transfection, filtered and applied to HeLaM cells depleted of HLA-A, -B, -C (HLA-ABC ^KO^) ^50^ and transduced with HLA-A*68:02 ^17^. Supernatant volume was increased to 2mL with DMEM and supplemented with 8μg/mL polybrene (Sigma Aldrich, TR-1003).

### Peptide exchange assay

HeLaM ABC ^KO^ reconstituted with HLA-A*68:02 and expressing the TAPBPR variants were incubated with the indicated concentrations of TAMRA-labelled peptide (ETVSK ^TAMRA^QSNV) in Opti-MEM (Gibco, 51985026) for 15 min at 37°C. Subsequently, cells were washed with PBS and detached with 0.05% trypsin-ethylenediaminetetraacetic acid (EDTA; Gibco, 25300054). Surface TAPBPR was stained with PeTe6 ^49^ conjugated in-house to Alexa Fluor 680 (Thermo Fisher, A20188), followed by analysis using CytoFlex S (Beckman-Coulter) and FlowJo v10 software. Peptide binding was assessed on TAPBPR-gated cells to normalise surface expression levels.

### Datasets

The immunopeptidomics datasets have previously been described ^9, 25^ and are available at the Dryad Digital Repository ^28, 51^.

## Results

### TAPBPR filters more peptides than it loads on HLA-A*68:02, -B*15:03 and -C*12:03

Although TAPBPR has been shown to preferentially bind HLA-A molecules, interactions with HLA-B and -C molecules have previously been observed ^9, 17^. To assess how TAPBPR impacts the peptide repertoire presented by each of the three allotypes, we re-examined a published immunopeptidomics dataset comparing wild-type (WT) HeLaM cells, which in the absence of IFN-γ treatment express negligible levels of TAPBPR, with cells overexpressing (OE) TAPBPR ^9^. The total number of unique peptide sequences, i.e. the repertoire diversity, was decreased in TAPBPR OE cells compared to WT cells ( Figure 1A). These findings were complemented by comparing the peptide repertoire eluted from WT HeLaM cells, treated with IFN-γ to induce endogenous TAPBPR expression, to IFN-γ-treated cells in which TAPBPR had been depleted (knock-out [KO] clone) ^9^. TAPBPR-depleted cells showed an increased repertoire diversity compared to the WT cells, confirming that endogenous TAPBPR also restricts the HLA class I repertoire on HeLaM cells ( Figure 1B).

We further assigned the peptides identified from these datasets into three different categories. Peptides detected in both WT and OE TAPBPR cells were considered TAPBPR-independent ( Figure 1A). Peptides unique to untreated WT cells were presumed to be dissociated or prevented from loading (i.e. filtered) by TAPBPR as they were only detected in cells expressing negligible levels of TAPBPR ( Figure 1A). Conversely, peptides unique to TAPBPR OE cells were assumed to require TAPBPR for loading onto HLA class I ( Figure 1A). Similar categories were assigned in the comparison of IFN-γ-treated TAPBPR KO and WT cells ( Figure 1B). Interestingly, in addition to peptide filtering, TAPBPR also appeared to assist in peptide loading on HLA class I.

The peptides dependent on TAPBPR for filtering or loading were subsequently assigned to HLA-A*68:02, -B*15:03 and -C*12:03. Both OE and endogenous TAPBPR filters more peptides than it loads, i.e. restricts the peptide repertoire, on all three allotypes expressed by HeLaM cells ( Figure 1C-D). While TAPBPR-mediated restriction of the peptide repertoire on HLA-A*68:02 and -B*15:03 had previously been identified ^9^, the role of TAPBPR in restricting the repertoire presented on HLA-C*12:03 was not originally characterised.

### The TAPBPR editing loop contributes to peptide loading as well as peptide filtering

The role of the TAPBPR editing loop in peptide selection had previously been assessed by establishing the peptide repertoire from HeLaM TAPBPR KO cells transduced to express wild-type TAPBPR (TAPBPR ^WT^), TAPBPR with mutated residues of the loop domain (TAPBPR ^øloop^), or TAPBPR ^øloop^ in which the leucine had been reconstituted at position 30 (TAPBPR ^øG30L^) ( Table 1) ^25^. Using two-way comparisons between the TAPBPR ^WT^ with either TAPBPR ^øloop^ or TAPBPR ^øG30L^, a key role L30 in mediating peptide dissociation from HLA class I was revealed ^11^. This was observed in two independent datasets which differed in the recovery period post-trypsin harvest (addressed in more detail below). As additional mechanisms for the TAPBPR editing loop in peptide editing were proposed more recently ^26^, reanalysis of these datasets could shed further light into how the editing loop functions in the peptide selection.

**Table 1.** TAPBPR variants with mutations in the K22-D35 editing loop. Modified residues are highlighted in red. TAPBPR variants were transduced into TAPBPR KO HeLaM cells.

TAPBPR variant	Sequence editing loop
WT	KDGAHRGA **L**ASSED
øloop	AAGGSGGG **G**SGGAA
øG30L	AAGGSGGG **L** GGGAA

In our reassessment, the HLA class I peptides shared between cells expressing TAPBPR ^WT^ or TAPBPR ^øloop^ were considered to represent peptides that were not dependent on TAPBPR and/or the editing loop ( Figure 2A). Peptides unique to cells expressing TAPBPR ^WT^ were assumed to require the editing loop to be loaded on HLA class I since they were not eluted from cells expressing TAPBPR ^øloop^. Conversely, peptides unique to cells expressing TAPBPR ^øloop^ were presumed to be filtered by the TAPBPR ^WT^ editing loop. While it was previously highlighted that the editing loop was involved in peptide filtering ^24, 25^, our reanalysis showed that it also contributes to peptide loading onto HLA class I molecules. Slightly more peptides were filtered than loaded by the editing loop ( Figure 2A).

Direct comparisons of the immunopeptidomes from cells expressing TAPBPR ^øloop^ or TAPBPR ^øG30L^ were not previously established. When we performed this analysis, we observed that the increase in repertoire size caused by mutating the residues in the editing loop (TAPBPR ^øloop^) was reverted by reconstitution of L30 (TAPBPR ^øG30L^, Figure 2B). Thus, L30 appears to be sufficient to restrict the peptide repertoire diversity on the HLA class I molecules expressed by HeLa cells.

### HLA class I is in a peptide receptive state with either TAPBPR ^WT^ or TAPBPR ^øloop^

Peptide filtering by L30 likely requires binding of this residue in the HLA class I F pocket and is presumably dependent on opening of the peptide binding groove ^22, 24, 25^. It was previously shown that TAPBPR causes HLA class I molecules to enter an open conformation and this conformational change was hypothesised to be mediated by the editing loop ^21, 22^. However, this hypothesis contrasts our observation that L30 can mediate repertoire restriction in absence of the other loop residues ( Figure 2B). Therefore, all-atom MD simulations were conducted to test whether the peptide binding groove was open in absence of the wild-type editing loop residues. In line with our immunopeptidomics results, TAPBPR ^øloop^ retained its ability to open the peptide binding cleft of peptide-deficient HLA-A*68:02 ( Figure 2C-D [0-15ns]). While TAPBPR ^WT^ allowed the HLA-A*68:02 groove to remain open during the 100ns simulation, the peptide binding cleft narrows with TAPBPR ^øloop^ ( Figure 2D).

The peptide receptive state of the HLA-A*68:02 peptide binding groove was assessed in a peptide exchange assay where the surface expression of OE TAPBPR was utilised to mediate peptide exchange on surface HLA class I molecules ^11, 25^. Using this assay, we previously showed that the TAPBPR editing loop was essential to mediate efficient peptide dissociation from HLA-A*68:02 ^25^. Here, we assessed the ability of TAPBPR editing loop variants to load exogenous fluorescently labelled peptide on HLA-A*68:02. Wild-type HeLaM cells that do not express TAPBPR at the cell surface were used as a negative control. HLA-A*68:02 associated with TAPBPR ^øloop^ was more peptide receptive compared to when it was bound to TAPBPR ^WT^ and TAPBPR ^øG30L^ ( Figure 2E).

MD simulations showed that opening of the HLA-B*15:03 and C*12:03 peptide binding groove was similar with TAPBPR ^WT^ or TAPBPR ^øloop^ ( Figure 2F–G). Taken together, while the editing loop allows the HLA-A*68:02 peptide binding groove to remain open for a prolonged period, other regions of TAPBPR are likely to also contribute to the initial opening of the HLA class I peptide binding groove.

### TAPBPR editing loop-dependent peptide filtering is predominantly mediated by L30

The previous immunopeptidomics publication only compared cells expressing TAPBPR ^WT^ to either TAPBPR ^øloop^ or TAPBPR ^øG30L^-expressing cells ^25^. To further dissect the role of the editing loop in shaping the diversity of the HLA class I peptide repertoire, we expanded this analysis by performing a three-way comparison of peptides eluted from cells expressing TAPBPR ^WT^, TAPBPR ^øloop^ and TAPBPR ^øG30L^. The peptides filtered by the editing loop (unique to TAPBPR ^øloop^ compared to TAPBPR ^WT^) were further subdivided by their presence or absence in the repertoire eluted from cells expressing TAPBPR ^øG30L^ ( Figure 3A). Peptides unique to TAPBPR ^øloop^ (compared to TAPBPR ^WT^) and not identified in TAPBPR ^øG30L^ (n=295) were considered dependent on L30 for filtering ( Figure 3A). The remaining 133 peptides in the unique to TAPBPR ^øloop^ subset, were assumed to be filtered in a TAPBPR editing loop-dependent but L30-independent manner ( Figure 3A). The peptides in these subgroups were assigned to the HLA class I molecules expressed by HeLaM, which revealed that peptide filtering on all three HLA class I allotypes was mostly L30-dependent ( Figure 3B).

### L30 is typically not sufficient to mediate editing loop-dependent peptide loading

Next, the role of the editing loop in peptide loading was further assessed using the peptides unique to TAPBPR ^WT^ (compared to TAPBPR ^øloop^) ( Figure 3A). Peptides unique to TAPBPR ^WT^ and not identified in TAPBPR ^øG30L^ (n=198) were considered peptides where L30 alone was not sufficient to load them onto HLA class I, i.e. additional or other residues in the editing loop contribute to peptide loading. For the remaining 147 peptides in the subset unique to TAPBPR ^WT^, L30 was presumed sufficient for HLA class I loading ( Figure 3A). Most peptides loaded onto HLA-A*68:02 and -B*15:03 required the TAPBPR ^WT^ editing loop, suggesting that L30 alone was not sufficient for loading ( Figure 3C). This was not the case for peptides assigned to HLA-C*12:03, for which L30 seemed sufficient to load most peptides ( Figure 3C). However, the difference and the peptide number are small. Taken together, in addition to filtering, the editing loop seems to contribute to peptide loading, particularly on HLA-A*68:02 and B*15:03, and typically required additional or other residues than L30.

### The recovery period post-trypsinisation impacts the immunopeptidome

It was previously reported that OE TAPBPR traffics to the cell surface where it can perform peptide exchange on surface expressed HLA class I ^11^. Therefore, two datasets were originally established with the TAPBPR editing loop mutants in which cells were harvested immediately after trypsinisation (no recovery) or incubated at 37°C in media for 30min post-trypsinisation to permit the OE surface TAPBPR to perform peptide editing on surface HLA class I ^25^. Curiously, when allowing the surface expressed TAPBPR to edit on HLA class I using a 30min recovery period after trypsinisation, the editing loop-dependent filtering seemed to be relatively less dependent on L30 ( Figure 3D–E). Conversely, TAPBPR editing loop-dependent loading seems more dependent on the additional/other residues than L30 compared to directly after trypsinisation ( Figure 3D/F).

## Discussion

Here, immunopeptidomic datasets were reanalysed in-depth and HLA class I molecular dynamics simulations were performed to provide further insight into the mechanism of action of TAPBPR-mediated HLA class I peptide exchange. TAPBPR was previously shown to preferentially bind and edit the repertoire on HLA-A, although interactions with and editing on HLA-B molecules has also been described ^9, 17^. We showed that TAPBPR can shape the peptide repertoire on HLA-A, -B and interestingly also on HLA-C molecules. The previously observed preference of TAPBPR for HLA-A over HLA-B or -C ^17^ may reflect variations in the kinetics of TAPBPR binding. Since peptide binding to MHC-I drives TAPBPR dissociation by a negative allostery release cycle ^52^, a stronger TAPBPR:HLA-A interaction may persist longer as it takes a high affinity peptide to outcompete TAPBPR. Alternatively, TAPBPR may remain bound until UGT1 has mediated recycling of the peptide-receptive HLA-A back to the PLC. This recycling was more difficult to detect for HLA-B or -C molecules ^12^. Furthermore, HLA-C is expressed at lower levels compared to HLA-A or -B ^53^, further complicating detection of interactions. The impact of TAPBPR on the peptide repertoire eluted from HLA-C indicates that TAPBPR may edit on a wider variety of allotypes than initially thought, which would be of interest to explore in future functional assays.

The three HLA class I alleles expressed by HeLaM cells all accommodate hydrophobic amino acids in their F pocket. This was shown to be a key feature for TAPBPR editing loop, and specifically L30, -mediated peptide exchange ^25^. There are currently two main hypotheses for the role that the TAPBPR editing loop plays in peptide exchange. First, the TAPBPR editing loop was suggested to act as a *scoop loop* and function as an internal peptide surrogate, with L30 as the critical residue ( *leucine lever*), thereby filtering the peptide repertoire ^22, 24, 25^. Secondly, the editing loop may hover above the peptide groove and act as a *peptide trap* ^26^. Interestingly, immunopeptidomics using cells expressing the TAPBPR editing loop mutants suggests that the K22-D35 domain is involved both in peptide filtering and loading on all three HLA class I allotypes expressed by HeLa cells.

With both TAPBPR ^WT^ and TAPBPR ^øG30L^ mediating restriction of the peptide repertoire compared to TAPBPR ^øloop^, we hypothesised that TAPBPR allows the HLA class I peptide binding groove to adopt an open state independently of the editing loop to facilitate peptide exchange. Alternatively, L30 may be involved in opening of the HLA class I peptide binding cleft followed by peptide dissociation. All-atom MD simulations showed that TAPBPR ^øloop^ retained its ability to open the α2-1 helix of peptide-deficient HLA-A*68:02, while the TAPBPR ^WT^ editing loop held HLA-A*68:02 in an open state for a prolonged period. Regions such as the *jack hairpin*, which was previously be reported to interact with the α2-1 helix and cause a partial downward shift of the HLA-I peptide binding groov ^20, 22^, likely also contribute to the opening of the HLA class I peptide binding groove. It is important to note that our MD simulation results did not assess the role of the TAPBPR editing loop in opening of the peptide binding cleft with a peptide bound.

TAPBPR ^øloop^ allowed for more peptide binding than TAPBPR ^WT^ and TAPBPR ^øG30L^ in an HLA-A*68:02 peptide loading assay, indicating that the groove is open long enough for peptide loading. Additionally, these results indicate that the K22-D35 editing loop, and likely L30 in particular, increases the threshold for peptide binding. The reanalysis of immunopeptidomics datasets shows that the editing loop and specifically L30 are involved in TAPBPR-mediated peptide filtering. Data from Ilca *et al*. suggests that L30 binds the F pocket of HLA class I molecules, outcompeting the bound peptide and causing its dissociation ^25^. It appears that residues outside of the editing loop are involved in the opening of the peptide binding groove, however, this mechanism alone may not be sufficient to mediate peptide filtering. Entry of L30 into the F pocket permitted by opening of the peptide binding groove is likely required for the peptide filtering ability of TAPBPR. While the L30 residue was typically sufficient to mediate filtering, peptide loading typically required other residues in the TAPBPR editing loop. These data complement published MD simulations showing that R27 in the TAPBPR editing loop may draw a peptide into the F pocket ^23^. Taken together, our reanalysis suggests the TAPBPR editing loop is multifunctional and could be involved in peptide selection by both the previously proposed *scoop loop/leucine lever* and *peptide trap* models, with L30 as the critical residue for peptide filtering and additional or other residues in the editing loop sustaining the HLA class I open state and assisting with peptide loading.

Immunopeptidomics of cells expressing TAPBPR editing loop variants was carried out in duplicate ^25^. Cells were lysed immediately after harvesting the cells (no recovery) or cells were incubated 30min at 37°C post-trypsinisation, allowing OE surface TAPBPR to perform HLA class I peptide editing ^25^. The overall conclusion of the effect of the TAPBPR editing loop, and specifically L30, remain the same regardless of the recovery period. However, by adding the recovery period, TAPBPR editing loop-mediated filtering seemed less dependent on L30 whereas loading seemed more dependent on the editing loop. The underlying mechanism for this phenomenon is not clear. Although it has previously been suggested that HLA class I surface levels are not altered by trypsin-treatment of the cells ^54^, trypsin may disrupt the HLA class I immunopeptidome or the TAPBPR:HLA class I interaction at the plasma membrane. The relative proportion of TAPBPR-mediated peptide exchange occurring in different subcellular compartment post-trypsin recovery, i.e. at the cell surface versus intracellular, may have caused a shift in the peptidome. The use of suspension cells that do not require trypsinisation as part of the harvesting process may be useful in future immunopeptidomics experiments.

We have previously shown that soluble recombinant TAPBPR can be utilised to display immunogenic peptides on cell surface-expressed HLA class I which triggers T-cell-mediated killing of target cells ^11, 55^. This reveals the translational potential to use TAPBPR as an immunotherapeutic. Insight into TAPBPR-mediated peptide editing may aid rationale design and improve efficiency of TAPBPR-based therapeutic candidates on polymorphic HLA class I molecules. For example, insight into the mechanism of the TAPBPR editing loop may allow us to improve HLA class I peptide binding or alter TAPBPR activity to HLA class I molecules on which the editing loop does not work naturally. Increased fundamental understanding of the mechanisms governing TAPBPR-mediated peptide exchange could expand the range of HLA class I allotype and therefore potentially the range of patients that may be responsive to TAPBPR-based therapies.

## Data Availability

The immunopeptidomics datasets used in this study are available from: Dryad: Data from: TAPBPR alters MHC class I peptide presentation by functioning as a peptide exchange catalyst. https://doi.org/10.5061/dryad.487j9 ^28^. Dryad: Data from: TAPBPR mediates peptide dissociation from MHC class I using a leucine lever. https://doi.org/10.5061/dryad.p5k0156 ^51^. Data are available under the terms of the Creative Commons Zero "No rights reserved" data waiver (CC0 1.0 Public domain dedication). Files related to the flow cytometry experiments and MD simulations in this study are available from: Figshare: Data related to Reanalysis of Immunopeptidomics Datasets Provides Mechanistic Insight into TAPBPR-Mediated Peptide Editing on HLA-A, -B and -C Molecules - Version 2. doi: 10.6084/m9.figshare.30517928 Data are available under the terms of the CC BY 4.0 licence.
