# Supplementary material for: Reanalysis of Immunopeptidomics Datasets Provides Mechanistic Insight into TAPBPR-Mediated Peptide Editing on HLA-A, -B and -C Molecules
Source: Wellcome Open Res. 2025 Nov 25;9:113. Originally published 2024 Mar 1. [Version 2] doi: 10.12688/wellcomeopenres.20738.2 (PMC11126903; doi:10.12688/wellcomeopenres.20738.2)
Supplement: Peer review history

# Reviewer report: Reviewer response for version 2

**DOI:** 10.21956/wellcomeopenres.27828.r140782

**Published:** 2025-12-23

**Authors:** Rui Chen

**Affiliations:** 1 National Research Council Canada, Ottawa, Ontario, Canada

The authors have addressed the issues from the reviewer and revised the manuscript accordingly. No further comments are made to the revised manuscript.

Is the work clearly and accurately presented and does it cite the current literature?

Yes

If applicable, is the statistical analysis and its interpretation appropriate?

No

Are all the source data underlying the results available to ensure full reproducibility?

Partly

Is the study design appropriate and is the work technically sound?

Yes

Are the conclusions drawn adequately supported by the results?

Partly

Are sufficient details of methods and analysis provided to allow replication by others?

No

Reviewer Expertise:

proteomics, immunopeptidomics, systems biology, mass spectrometry

I confirm that I have read this submission and believe that I have an appropriate level of expertise to confirm that it is of an acceptable scientific standard.

# Reviewer report: Reviewer response for version 2

**DOI:** 10.21956/wellcomeopenres.27828.r140784

**Published:** 2025-12-08

**Authors:** Pietro Crivello

**Affiliations:** 1 University Hospital Essen, Essen, Germany

I have no additional comments to do at this stage and approve the paper at this present version.

Is the work clearly and accurately presented and does it cite the current literature?

Yes

If applicable, is the statistical analysis and its interpretation appropriate?

Not applicable

Are all the source data underlying the results available to ensure full reproducibility?

Yes

Is the study design appropriate and is the work technically sound?

Partly

Are the conclusions drawn adequately supported by the results?

Partly

Are sufficient details of methods and analysis provided to allow replication by others?

Partly

Reviewer Expertise:

NA

I confirm that I have read this submission and believe that I have an appropriate level of expertise to confirm that it is of an acceptable scientific standard.

# Reviewer report: Reviewer response for version 1

**DOI:** 10.21956/wellcomeopenres.22949.r78735

**Published:** 2024-05-25

**Authors:** Rui Chen

**Affiliations:** 1 National Research Council Canada, Ottawa, Ontario, Canada

The manuscript submitted by Altenburg et al. described a novel approach to study the role of the TAPBPR editing loop in opening the HLA-I peptide binding groove by using molecular dynamics simulation with immunopeptidomics data from previous studies that generated at different conditions of Hela cells, i.e., wild-type, depleted TAPBPR and overexpressed of TAPBPR or TAPBPR K22-D35 mutant. Although this study delivered some insights into the relationship between TAPBPR editing loop and antigen presentation of different HLA alleles, there is serious concern about the immunopeptidomics dataset that the authors used, which need to be addressed.

 Detailed comments

 1  The two datasets used in this study were generated in 2013 and 2018, respectively, by a relatively older mass spectrometer compared to that has been used in recent publications, which leads to the lack of depth towards the coverage of whole immunopeptidome. There was no mention of technical or biological replicates from these two studies which compromised the statistical significance of the current study. Also, the immunopeptidomics data were not quantitative. It might be difficult to repeat the experiments, however, re-analysis of the mass spectrometric data with relative quantification (LFQ) is needed.

 2  The analysis of the two datasets needs to be harmonized as different protein database search engines were used and different criteria of peptides identification filtering were used. After the generation of peptides identification, same tools need to be used to predict the binding affinity of identified peptides to HLA alleles. It is recommended that the author to use the lasted version of netPanMHC( https://services.healthtech.dtu.dk/services/NetMHCpan-4.1/). It is not clear what parameters were used and the authors need to add more details about the HLA-I peptides analysis.

Is the work clearly and accurately presented and does it cite the current literature?

Yes

If applicable, is the statistical analysis and its interpretation appropriate?

No

Are all the source data underlying the results available to ensure full reproducibility?

Partly

Is the study design appropriate and is the work technically sound?

Yes

Are the conclusions drawn adequately supported by the results?

Partly

Are sufficient details of methods and analysis provided to allow replication by others?

No

Reviewer Expertise:

proteomics, immunopeptidomics, systems biology, mass spectrometry

I confirm that I have read this submission and believe that I have an appropriate level of expertise to confirm that it is of an acceptable scientific standard, however I have significant reservations, as outlined above.

# Response

**Published:** 2025-11-03

**Authors:** Arwen Altenburg

**Affiliations:** Pathology, University of Cambridge, Cambridge, England, UK

**1. The two datasets used in this study were generated in 2013 and 2018, respectively, by a relatively older mass spectrometer compared to that has been used in recent publications, which leads to the lack of depth towards the coverage of whole immunopeptidome. There was no mention of technical or biological replicates from these two studies which compromised the statistical significance of the current study. Also, the immunopeptidomics data were not quantitative. It might be difficult to repeat the experiments, however, re-analysis of the mass spectrometric data with relative quantification (LFQ) is needed.**

**Response:** *We agree with the reviewer that newer mass spectrometer instruments show substantially improved sensitivity and quality of immunopeptidomics datasets. We also agree with the reviewer on the value of semi-quantitative analysis and are excited to explore the potential of the more sensitive approach in our future work. However, for the current study we have reanalysed datasets that were previously generated according to the standards at the time of publication. The dataset related to Figure 1 was measured with three technical replicates using a DDA-method, and therefore a semi-quantitative analysis is not feasible. Furthermore, due to the well-established technical limitations of semi-quantitative analysis of LC-MS/MS data, a retrospective LFQ analysis of differently acquired datasets could not yield unbiased significant results.* *While current methods are improved, we believe the older publicly available datasets remain valuable. Our in-depth re-analysis has highlighted previously undetected findings that provide further insight into the mechanism of action of TAPBPR-mediated HLA class I peptide exchange that we feel are important to share with others in the field.* *Further information on the biological and technical replicates in available in response to the reviewer report of Pietro Crivello (question 1). A statement on technical replicates has been added to the Methods section.*     

**2.  The analysis of the two datasets needs to be harmonized as different protein database search engines were used and different criteria of peptides identification filtering were used. After the generation of peptides identification, same tools need to be used to predict the binding affinity of identified peptides to HLA alleles. It is recommended that the author to use the lasted version of netPanMHC(https://services.healthtech.dtu.dk/services/NetMHCpan-4.1/). It is not clear what parameters were used and the authors need to add more details about the HLA-I peptides analysis.**

**Response:** *As we re-analysed previously published datasets, HLA binding predictions were performed using the state-of-the-art methods of the time. Information regarding HLA assignments has been added to the Methods section of the manuscript.* *Dataset related to Figure 1: binder prediction was conducted with the online version of NetMHC-3.0.* *Dataset related to Figures 2&3: HLA annotation was performed using NetMHCpan-4.0 with a percentile rank threshold of 2%.*

# Reviewer report: Reviewer response for version 1

**DOI:** 10.21956/wellcomeopenres.22949.r82626

**Published:** 2024-05-22

**Authors:** Priyanka Rana

**Affiliations:** 1 Case Western Reserve University, Cleveland, Ohio, USA

The article by Altenburg et al, investigates the molecular mechanisms by which the TAPBPR molecule influences peptide selection and exchange on major histocompatibility class I (MHC-I) molecules, specifically focusing on human leukocyte antigen (HLA)-I. MHC-I molecules are critical for immune surveillance, presenting peptides on the cell surface to allow the immune system to detect infected or malignant cells. TAPBPR, along with tapasin, is known to optimize the peptide repertoire presented by MHC-I, but the exact mechanisms have been unclear.

 The study utilizes in-depth re-analyses of immunopeptidomics datasets and molecular dynamics simulations to explore how TAPBPR edits the peptide repertoire on HLA-A, -B, and -C allotypes. The findings indicate that the editing loop of TAPBPR is not essential for opening the HLA-I peptide binding groove but plays a significant role in peptide repertoire restriction, particularly through the leucine at position 30 (L30). This residue is crucial for both peptide dissociation and loading, suggesting that TAPBPR can mediate peptide exchange via a combination of the scoop loop and peptide trap mechanisms. The introduction effectively sets the stage by explaining the role of MHC-I in immune surveillance and the importance of TAPBPR in peptide editing.

 The background information is well-articulated, providing a clear context for the study.

 The use of re-analysis of immunopeptidomics datasets and molecular dynamics simulations is appropriate and robust for studying the mechanisms of TAPBPR. These methods are well-suited to uncovering detailed molecular interactions.

 The methods section could benefit from a more detailed explanation of the specific datasets and simulation parameters used. For instance, the criteria for dataset selection and the specifics of the molecular dynamics simulations (e.g., force fields, simulation times) should be clearly stated.

 The discussion effectively integrates the results with existing knowledge, providing a cohesive understanding of TAPBPR's role in peptide editing. The unification of the scoop loop and peptide trap mechanisms is a particularly valuable contribution to the field. The discussion could be expanded to address potential limitations of the study. For example, the reliance on computational simulations and re-analysis of existing datasets might limit the generalizability of the findings. Discussing these limitations and suggesting future experiments to validate the results would enhance the discussion.

 The conclusions are well-supported by the data and provide a clear summary of the key findings. The identification of L30 as a critical residue for peptide exchange is particularly noteworthy. Expand the conclusions to include a brief discussion on the potential therapeutic implications of manipulating TAPBPR-HLA-I interactions, which could be of interest for developing novel immune-based therapies.

Is the work clearly and accurately presented and does it cite the current literature?

Yes

If applicable, is the statistical analysis and its interpretation appropriate?

Yes

Are all the source data underlying the results available to ensure full reproducibility?

Yes

Is the study design appropriate and is the work technically sound?

Yes

Are the conclusions drawn adequately supported by the results?

Yes

Are sufficient details of methods and analysis provided to allow replication by others?

Yes

Reviewer Expertise:

Immuno-oncology,, Hematology, Oncology, TNBC

I confirm that I have read this submission and believe that I have an appropriate level of expertise to confirm that it is of an acceptable scientific standard.

# Response

**Published:** 2025-11-03

**Authors:** Arwen Altenburg

**Affiliations:** Pathology, University of Cambridge, Cambridge, England, UK

**1. The methods section could benefit from a more detailed explanation of the specific datasets and simulation parameters used. For instance, the criteria for dataset selection and the specifics of the molecular dynamics simulations (e.g., force fields, simulation times) should be clearly stated.**

**Response:** *This manuscript includes a re-analysis of immunopeptidomics datasets that have previously been obtained and described to assess TAPBPR function. No alternative datasets were available to choose from. A detailed explanation of the publicly available datasets is available in the original publications, which are referenced throughout the manuscript, including in the Methods section and Data availability statement.* *Additional information on molecular dynamics simulations such as simulation times have been described in the Methods section. *    

**2. The discussion effectively integrates the results with existing knowledge, providing a cohesive understanding of TAPBPR's role in peptide editing. The unification of the scoop loop and peptide trap mechanisms is a particularly valuable contribution to the field. The discussion could be expanded to address potential limitations of the study. For example, the reliance on computational simulations and re-analysis of existing datasets might limit the generalizability of the findings. Discussing these limitations and suggesting future experiments to validate the results would enhance the discussion.**

**Response:** *We thank the reviewer for their comments and acknowledge the limitation of computational simulations and re-analysis of previously published datasets. We added experimental data that aligns with the observations from the MD simulations as explained above (Pietro Crivello, question 4).* *Additionally, future experiments have been suggested in the Discussion section. For example, the use of suspension cells that do not require trypsinisation as part of the harvesting process may be useful in future immunopeptidomics experiments. Furthermore, future (immunopeptiomics) work may investigate TAPBPR function and mechanism of action on a wider selection of HLA class I molecules, other than the three allotypes expressed by HeLa cells.*  

**3. The conclusions are well-supported by the data and provide a clear summary of the key findings. The identification of L30 as a critical residue for peptide exchange is particularly noteworthy. Expand the conclusions to include a brief discussion on the potential therapeutic implications of manipulating TAPBPR-HLA-I interactions, which could be of interest for developing novel immune-based therapies.**

**Response:** *We appreciate the reviewer’s comment and are excited about exploring the potential of TAPBPR-based therapeutics as an avenue of future research. However, we are reluctant to expand the conclusions described in the manuscript further as the work constitutes a re-analysis of publicly available datasets and MD simulations. As indicated by the reviewer in the previous point (2), we are mindful of this limitation of the described work and do not want to overstate the conclusions. A description of the therapeutic potential of TAPBPR is available in the last paragraph of the Discussion section to place the work into context. Additionally, this topic has been previously reviewed in Ilca et al., 2020, Current Opinion in Immunology.*

# Reviewer report: Reviewer response for version 1

**DOI:** 10.21956/wellcomeopenres.22949.r78734

**Published:** 2024-05-02

**Authors:** Pietro Crivello

**Affiliations:** 1 University Hospital Essen, Essen, Germany

In this manuscript, the authors provide interesting new insights in the mechanism of interaction between HLA class I molecules and the peptide editor TAPBPR.

 Published immunopeptidomics data and molecular dynamics simulations were used to expand the understanding of this process, in particular investigating the role of the flexible loop K22-D35 of TAPBPR and the residue Leucine at position 30.

 The results shown here seem to concile two reported mechanims of action invoking TAPBPR as both filter and loading helper for HLA class I peptides.

**The authors should also consider the following critical points:**

- Are the presented peptide repertoires obtained from single or multiple measurements? In the second case, how much is the technical/biological reproducibility? Especially, are the peptides edited by TAPBPR reproducibly found in their corresponding experimental condition?
-  A large amount of peptides (n=1398) could be found in TAPBPR KO and OE cell lines. However, within this group many peptides might still be affected by TAPBPR expression in quantitative terms. Peptides being more or less presented in the repertoire when TAPBPR is also present. Comparison of the MS1 peak intensities might help to further describe the function of TAPBPR in quantitative terms.
- The authors used netMHC to predict whether the identified peptides were either HLA-A,B or C binders, in order to dissect the role of TAPBPR across different loci. NetMHC can also be used to estimate the affinity of the peptides to those molecules. Is there any difference in affinity between peptides filtered/loaded by TAPBPR?
- In the molecular dynamics experiment, the authors suggest that absence of editing loop in TAPBPR does not affect the ability of the molecule to open the peptide binding cleft. Up to 2 Angstrom difference is reported. However, how much is the cleft opening if the same modelling is performed with TAPBPR WT? Additionally, the results are reported for the allotype HLA-A2, would it be possible to perform the same modelling for the allotypes investigated with immunopeptidomics data (A*68:02, B*15:03 and C*12:03)?
- Are HLA class I molecules expressed at similar level in all cell lines investigated?
- In figure 2, it could be useful to compare TAPBPR-WT versus G30L variant.
- Is there a specific reason to justify the use of HeLaM in comparison to other cell lines, including the original HeLa cell line?

Is the work clearly and accurately presented and does it cite the current literature?

Yes

If applicable, is the statistical analysis and its interpretation appropriate?

Not applicable

Are all the source data underlying the results available to ensure full reproducibility?

Yes

Is the study design appropriate and is the work technically sound?

Partly

Are the conclusions drawn adequately supported by the results?

Partly

Are sufficient details of methods and analysis provided to allow replication by others?

Partly

Reviewer Expertise:

Immunogenetics, Hematopoietic Cell Transplantation, Alloreactivity, Immunopeptidomics

I confirm that I have read this submission and believe that I have an appropriate level of expertise to confirm that it is of an acceptable scientific standard, however I have significant reservations, as outlined above.

# Response

**Published:** 2025-11-03

**Authors:** Arwen Altenburg

**Affiliations:** Pathology, University of Cambridge, Cambridge, England, UK

**1. Are the presented peptide repertoires obtained from single or multiple measurements? In the second case, how much is the technical/biological reproducibility? Especially, are the peptides edited by TAPBPR reproducibly found in their corresponding experimental condition?**

**Response:** *We thank the reviewer for their questions. A statement regarding repeated measurements has been added to the Methods section of the manuscript. In brief:* *Dataset related to Figure 1 - LC-MS/MS measurements were conducted with three technical replicates applying a data-dependent acquisition (DDA)-method.* *Dataset related to Figures 2&3 - peptide elution experiments were performed in five technical replicates for all cell lines. Technical reproducibility of LC-MS/MS measurements is described in Figure 5—figure supplement 1 in the original paper describing the dataset (Ilca et al., 2018, eLife). >70-75% of peptides were measured in 2 or more replicates (out of 5).*    

**2. A large amount of peptides (n=1398) could be found in TAPBPR KO and OE cell lines. However, within this group many peptides might still be affected by TAPBPR expression in quantitative terms. Peptides being more or less presented in the repertoire when TAPBPR is also present. Comparison of the MS1 peak intensities might help to further describe the function of TAPBPR in quantitative terms.**

**Response:** *We agree with the reviewer that it would be interesting to analyse if there is a change in abundance of HLA class I peptides in the presence or absence of TAPBPR. Unfortunately, we don’t have quantitative information available on the dataset published in 2015 comparing TAPBPR WT, KO and OE cell lines. As this dataset was measured with three technical replicates applying a DDA-method, a semi-quantitative analysis is not feasible. We plan to use semi-quantitative analysis on future datasets that we will obtain to further explore the impact of TAPBPR on peptide abundance.*    

**3. The authors used netMHC to predict whether the identified peptides were either HLA-A,B or C binders, in order to dissect the role of TAPBPR across different loci. NetMHC can also be used to estimate the affinity of the peptides to those molecules. Is there any difference in affinity between peptides filtered/loaded by TAPBPR?**

**Response:** *We agree with the reviewer that it would be interesting to assess the affinity between peptides that are filtered or loaded in the TAPBPR-dependent manner. In this publication, we re-analysed publicly available immunopeptidomics datasets investigating TAPBPR function. Unfortunately, the power of the dataset published in 2015 is not sufficient for a reliable comparison of predicted affinities. We appreciate the helpful suggestion for the analysis and will keep it in mind for future immunopeptidomics experiments.*    

4. In the molecular dynamics experiment, the authors suggest that absence of editing loop in TAPBPR does not affect the ability of the molecule to open the peptide binding cleft. Up to 2 Angstrom difference is reported. However, how much is the cleft opening if the same modelling is performed with TAPBPR WT? Additionally, the results are reported for the allotype HLA-A2, would it be possible to perform the same modelling for the allotypes investigated with immunopeptidomics data (A*68:02, B*15:03 and C*12:03)?

**Response:** We thank the reviewer for their suggestions and ran MD simulations on HLA-A*68:02, -B*15:03 and -C12:03. The results have been included in the manuscript (version 2, Figure 2). We also added experimental data to the Results section (version 2, Figure 2) showing that cells expressing TAPBPR ^øloop ^allow peptide loading at lower concentrations than TAPBPR ^WT ^or TAPBPR ^øG30L^. This confirms that even with the TAPBPR ^øloop ^bound, the HLA-A*68:02 groove can adopt a peptide receptive state.    

**5. Are HLA class I molecules expressed at similar level in all cell lines investigated?** **Response:** *A statement on validation of HLA class I levels has been added in the Methods section. In brief:* *Dataset related to Figure 1 - Figure 5B in the original paper describing the dataset (Hermann et al., 2015, PNAS) shows unchanged surface MHC class I levels in IFN-γ treated HeLa after TAPBPR depletion.* *Dataset related to Figures 2&3 - Figure 1E in the original paper describing the dataset (Ilca et al., 2018, PNAS) shows similar HLA-I levels in cells expressing either TAPBPR ^WT^, TAPBPR ^øloop^, or TAPBPR ^øG30L­­^.*    

**6. In figure 2, it could be useful to compare TAPBPR-WT versus G30L variant.**

**Response:** *The original paper describing the dataset includes a comparison of the immunopeptidome of cells expressing the TAPBPR ^WT ^or TAPBPR ^øG30L ^variants (Ilca et al., 2018, eLife). This revealed a key role of L30 in mediating peptide dissociation from HLA-I. As additional mechanisms for the TAPBPR editing loop in peptide editing were proposed since originally publishing the dataset, we reanalysed the datasets to shed further light into how the editing loop functions in the peptide selection. In our reanalysis, the TAPBPR ^øG30L^variant was used as a ‘rescue’ of the TAPBPR ^øloop^, showing that L30 appears to be sufficient to restrict the peptide repertoire diversity on the HLA-I molecules expressed by HeLa cells.*    

**7. Is there a specific reason to justify the use of HeLaM in comparison to other cell lines, including the original HeLa cell line?**

**Response:** HeLaM cells are a variant of the HeLa cell line that is more responsive to IFN (Tiwari et al., 1987). A statement on this has been included in the Methods section. Furthermore, HeLa cells have typically been used in previous publications on TAPBPR because these cells express HLA-A*68:02, which was identified as the strongest binder of TAPBPR (Ilca et al., 2019, Cell Reports).
